# The Use of a Fixed 50:50 Mixture of Nitrous Oxide and Oxygen to Reduce Lumbar Puncture-Induced Pain in the Emergency Department: A Randomized Controlled Trial

**DOI:** 10.3390/jcm11061489

**Published:** 2022-03-09

**Authors:** Mélissandre Nicot, Ludovic Miraillet, Bruno Pereira, Jean-Baptiste Bouillon-Minois, Julien Raconnat, Farès Moustafa, Jeannot Schmidt, Sophia Sickout-Arondo, Lise Bernard, Pierre Clavelou, Xavier Moisset

**Affiliations:** 1 Service des Urgences, CHU Clermont-Ferrand, F-63000 Clermont-Ferrand, France; melissandrenicot@gmail.com (M.N.); lmiraillet@chu-clermontferrand.fr (L.M.); jbbouillon-minois@chu-clermontferrand.fr (J.-B.B.-M.); jraconnat@chu-clermontferrand.fr (J.R.); fmoustafa@chu-clermontferrand.fr (F.M.); jschmidt@chu-clermontferrand.fr (J.S.); 2 Délégation Recherche Clinique & Innovation, Biostatistics Unit, CHU Clermont-Ferrand, F-63000 Clermont-Ferrand, France; bpereira@chu-clermontferrand.fr; 3 Physiological and Psychosocial Stress, LaPSCo, CNRS, Université Clermont Auvergne, F-63000 Clermont-Ferrand, France; 4 Unité de Nutrition Humaine, UNH, INRAE, Université Clermont Auvergne, F-63000 Clermont-Ferrand, France; 5 Neuro-Dol, Inserm, CHU Clermont-Ferrand, Université Clermont Auvergne, F-63000 Clermont-Ferrand, France; ssickoutarondo@chu-clermontferrand.fr (S.S.-A.); pclavelou@chu-clermontferrand.fr (P.C.); 6 ICCF, CNRS, Clermont Auvergne INP, CHU Clermont-Ferrand, Université Clermont Auvergne, F-63000 Clermont-Ferrand, France; l_bernard@chu-clermontferrand.fr

**Keywords:** pain, anxiety, lumbar puncture, emergency department, nitrous oxide, procedural pain

## Abstract

Lumbar puncture (LP) is stressful and often painful. We evaluated the efficacy of a fixed 50% nitrous oxide–oxygen mixture (50%N_2_O-O_2_) versus placebo to reduce immediate procedural pain and anxiety during LP performed in an emergency setting. We conducted a randomized controlled trial involving adults who needed a cerebrospinal fluid analysis in an emergency department. Patients were randomly assigned to inhale either 50%N_2_O-O_2_ or medical air. The primary endpoint, assessed using a numerical scale, was the maximum pain felt by the patient during the procedure and the maximum anxiety and satisfaction as secondary outcomes. Eighty-eight patients were randomized and analyzed (ITT). The maximal pain was 5.0 ± 2.9 for patients receiving air and 4.2 ± 3.0 for patients receiving 50%N_2_O-O_2_ (effect-size = −0.27 [−0.69; 0.14], *p* = 0.20). LP-induced anxiety was 4.7 ± 2.8 vs. 3.7 ± 3.7 (*p* = 0.13), and the proportion of patients with significant anxiety (score ≥ 4/10) was 72.7% vs. 50.0% (*p* = 0.03). Overall satisfaction was higher among patients receiving 50%N_2_O-O_2_ (7.4 ± 2.4 vs. 8.9 ± 1.6, *p* < 0.001). No serious adverse events were attributable to 50%N_2_O-O_2_ inhalation. Although inhalation of 50%N_2_O-O_2_ failed to reduce LP-induced pain in an emergency setting, it tended to reduce anxiety and significantly increased patient satisfaction.

## 1. Introduction

Lumbar puncture (LP) was described in the late 19th century [1]. Over 130 years later, this technical procedure, which gives direct access to the cerebrospinal fluid at the lumbar level, has become an indispensable diagnostic method in clinical practice, used daily in many hospital departments [2]. Nevertheless, the procedure is still stressful and possibly painful for most patients. Among hospital departments, the emergency department (ED) is among those in which LP is the most common, with an incidence of 0.8 per 100 admissions [2]. Although the LP technique is universal, the conditions under which LPs are performed and the indications are different between hospital departments and EDs. Indeed, EDs are overcrowded [3], resulting in potential delays in receiving prescriptions and therapeutics [4]. Furthermore, patients visiting an ED are often stressed [5] and in need of a rapid diagnosis. 

The use of a fixed 50% nitrous oxide/50% oxygen mixture (50%N^2^O-O_2_) has been proposed to make this procedure more comfortable for children [6,7], and a single study has been conducted on adults with a scheduled LP in a neurology setting [8]. In terms of EDs, this approach is also used empirically by certain emergency physicians as an extension of trauma indications [9], although there is no scientific proof for its efficacy in LP.

Nitrous oxide has been used for years for anesthesia during major surgery and its safety has been demonstrated [10], although it has some potential side effects (reduced parasympathetic tone, increased intracranial pressure, inactivation of vitamin B12 leading to potential hematologic and neurologic complications). As nitrous oxide is poorly soluble in blood, its analgesic effect and recovery after the procedure are rapid, with an elimination half-life of approximately 5 min [11]. Moreover, no additional staff or facilities are required for the safe administration or monitoring of the patient during or after the procedure, which is a major advantage for EDs. Adverse effects, although minor, have been reported to occur with a high frequency in some studies, and contradictory results have been obtained in terms of efficacy [12,13]. 

The purpose of this study was to evaluate the efficacy of 50%N_2_O-O_2_ inhalation versus placebo for reducing pain and anxiety during LP in adults in an emergency setting in a randomized control trial.

## 2. Materials and Methods

### 2.1. Study Design and Patients Selection

This double-blind, randomized, placebo-controlled trial was conducted at a single center (Clermont-Ferrand University Hospital, France). Patients visiting the ED and requiring an LP for any diagnostic indication were considered eligible if they were >18 years of age and never received nitrous oxide before. The exclusion criteria were a contraindication for nitrous oxide use (especially intracranial hypertension or already known B12 vitamin deficiency) [14], hemodynamic instability, a body-mass index >35 kg/m^2^, an ongoing pregnancy, confusion, a Mini Mental State Examination (MMSE) score < 24/30, and an inability to verbally communicate.

### 2.2. Administrative Issues

The clinical study was conducted in accordance with good clinical practice guidelines [15]. The protocol of the trial was approved by the appropriate ethics committee (CPP Sud-Ouest et Outremer 3, CNRIPH 18.07.27.84501). Patients gave written informed consent to participate in accordance with national regulations. The safety data and the conduct of the study were verified by an independent committee at the Clermont-Ferrand University Hospital. An independent data monitoring committee at Clermont-Ferrand University Hospital reviewed the conduct of the study and all safety data. The trial was registered on ClinicalTrials.gov under the reference NCT03941990. 

### 2.3. Randomization and Masking

Patients were assigned to the two treatment groups in accordance with a randomization list (random-size blocs) generated by an independent methodologist (BP). Patients who received 50%N_2_O-O_2_ were defined as the active treatment group and those that received medical air as the placebo group. Both gases (50%N_2_O-O_2_ and 22%O_2_–78%N_2_ medical air) were supplied by Air Liquide^®^ (Air Liquide Healthcare, Paris, France). We used block randomization stratified according to the history of LP, applying a 1:1 ratio. A dedicated nurse not participating in the administration of the product or the evaluation of the patient assigned the next number available on the list to each newly enrolled patient. The medicinal product investigated was administered by trained medical students with no role other than adapting the gas flow to ensure that there was a well-inflated reservoir bag attached to the non-rebreather mask (6 to 15 L per min). The detailed procedure was described in a previous study (gas bottles in identical boxes, a perfumed mask to make the sweet odor of N_2_O-O_2_ undiscernible) [8]. 

### 2.4. Procedures

An EMLA patch 5% cream (a eutectic mixture of local anesthetics containing 2.5% lidocaine and 2.5% prilocaine) was applied at the place envisaged to carry out the puncture, at least 1 h before the LP when possible, as this simple method has been shown to be an effective way to achieve analgesia for spinal puncture for all patients, avoiding the use of injectable lidocaine [16,17,18]. Inhalation began at a fixed rate of six liters per minute, 5 min before needle insertion, and was stopped just after needle removal. The sitting position and the requirement that the patient hold the mask ensured minimal or moderate sedation (conscious sedation). Two minutes after mask removal, the patient was asked to use two numerical rating scales (NRS) to assess first the maximum pain and then the maximum anxiety induced during the procedure. A third NRS was used to evaluate the overall satisfaction concerning the procedure. Patients were also asked about any adverse effects they had experienced.

### 2.5. Outcomes

The primary outcome was the maximum LP-induced pain during the procedure, assessed using an 11-point Likert scale between a minimum of 0 (no pain) and a maximum of 10 (the worst pain imaginable) recorded verbally between 2 and 3 min after the end of gas inhalation. The key secondary outcomes were the maximum LP-induced anxiety during the procedure, assessed using the same Likert scale as that used to assess LP-induced pain; and overall patient satisfaction, also assessed using a Likert scale ranging from 0 to 10, with a score of 10 indicating the highest possible satisfaction. Both pain and anxiety scores were recorded 2 to 3 min after discontinuation of the gas. Other prespecified secondary outcomes were based on the proportion of patients with pain scores ≥ 4/10 [19], anxiety scores ≥ 4/10, and satisfaction scores ≥ 9/10. Finally, the quality of the blinding and the cost of this additional procedure were evaluated. All adverse effects experienced were reported.

### 2.6. Statistical Analysis

The detection of a clinically relevant difference of two points [20] on an 11-point scale for the primary endpoint, with standard-deviation of 2.7, according to the previous study conducted in the neurology department [8], would require that we include 78 assessable patients—i.e., 39 per group—for a statistical power of 90% and a two-tailed type I error of 5%. In an emergency setting, incomplete data are possible, and we thus decided to include five more patients per group, leading to a total of 88 randomized patients.

Categorical data are expressed as the number of patients and the associated percentages, whereas continuous data are presented, according to the statistical distribution, as the mean and standard deviation or median and [interquartile range]. The assumption of normality was assessed using the Shapiro–Wilk test. 

The primary endpoint was compared between the randomization groups using Student’s *t*-test. The result is expressed as the effect-size (ES) and 95% confidence interval (95CI). The relationships between pain, age, and the number of LP attempts were analyzed using correlation coefficients (Pearson or Spearman, depending on the statistical distribution of the variables). The results are expressed using correlation coefficients (noted rho) and *p*-values. 

Other endpoints were compared between the randomization groups using chi-squared or Fisher exact tests for categorical variables (such as the proportion of patients with pain scores ≥ 4/10 and the proportion of patients with anxiety scores ≥ 4/10) and Student’s *t*-test or the Mann–Whitney test if the assumptions to apply the *t*-test were not met for quantitative variables (such as patient satisfaction). The assumption of homoscedasticity was checked using the Fisher-Snedecor test. The quality of blinding was also evaluated using the Bang blinding index [21]. The results are expressed using ESs, absolute differences, and the 95CIs. The number needed to treat (NNT) was also calculated.

Adjusted analyses were performed using robust random-effect Poisson generalized linear model regression with robust variance for binary outcomes. The covariates were selected according to univariate results and clinical relevance: age, BMI, number of LP attempts, and LP duration. The results are expressed using relative risks (RRs) and 95CIs.

Statistical analysis was performed on an intention-to-treat basis using Stata 16 software^®^ (StataCorp LP, College Station, TX, USA). The tests were two-tailed, with a type I error of 0.05. No correction for multiple testing was applied in the analysis of secondary outcomes, as they were considered to be exploratory. Missing data concerning the main outcomes (LP-induced pain, LP-induced anxiety, and overall satisfaction) were imputed using the mean value of the opposite group obtained in the per-protocol analysis (maximum bias). The per-protocol analysis is provided as a sensitivity analysis.

## 3. Results

**Figure 1:**
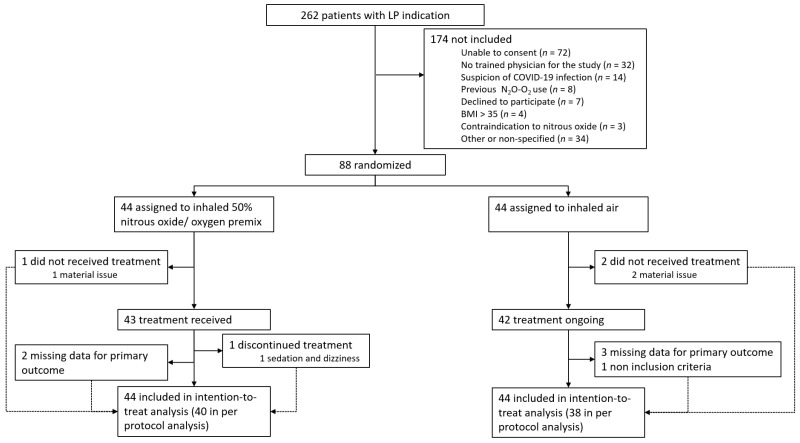
Trial profile. All randomly assigned patients were included in the intention-to-treat analysis.

Between 27 November 2019, and 5 October 2020, 262 patients had an LP in the ED of our university hospital, 88 of whom were randomized. Among the 88 randomized patients, 85 received the assigned treatment (43 for 50%N_2_O-O_2_ and 42 for air) and 79 files were fully completed (eligible for per-protocol analysis) (Figure 1). The demographic and baseline characteristics were similar for the two study groups, except for age, with older patients among those receiving air (Table 1).

The maximal pain was 5.0 ± 2.9 for the patients receiving air and 4.2 ± 3.0 for those receiving 50%N_2_O-O_2_ (ES = −0.27 [−0.69; 0.14], *p* = 0.20) (Table 2). Twenty-three patients receiving the active treatment (52.3%) reported an NRS score ≥ 4/10, versus 31 (70.5%) patients from the placebo group (*p* = 0.08). Thus, we needed to treat only 5.5 patients to prevent significant pain in one patient.

Similarly, the maximal LP-induced anxiety was 4.7 ± 2.8 vs. 3.7 ± 3.7 (ES = −0.32 [−0.74; 0.09], *p* = 0.13) (Table 2). However, the proportion of patients with significant anxiety (≥4/10) was lower in the active treatment group, with only 22 patients (50.0%) vs. 32 (72.7%) in the placebo group, leading the NNT to avoid one significant anxiety of 4.4 (*p* = 0.03). 

Overall satisfaction was higher among patients receiving 50%N_2_O-O_2_ (7.4 ± 2.4 vs. 8.9 ± 1.6, ES = −0.73 [−0.30; −1.16], *p* < 0.001) (Table 2). Indeed, 31 patients (70.5%) were very satisfied (≥9/10) in the active treatment group vs. 13 (29.6%) in the placebo group, leading to an NNT of 2.4 (*p* < 0.001). 

No serious adverse events were attributable to 50%N_2_O-O_2_ inhalation. One patient in the 50%N_2_O-O_2_ mixture group discontinued the inhalation treatment prematurely due to sedation and dizziness. Fifteen patients in the placebo group (39.5%) and twenty-six (65.0%) in the active treatment group described minor adverse effects (*p* = 0.024). Changes in sensory perception (vision, hearing) were the most frequently described (Table 3). 

Per-protocol analysis performed on the 79 patients with complete data provide the same results (Tables S1 and S2). 

Several factors were explored to identify predictive factors of pain induction by the procedure (Table 4). The duration of the LP and the number of attempts before a successful LP were significantly associated with pain intensity. Although age was not associated with pain, age and the number of LP attempts were associated (rho = 0.27; *p* = 0.02). The number of LP attempts was not influenced by the BMI, patients with a BMI ≤ 25 kg/m² requiring a mean of 1.7 ± 0.9 attempts vs. 2.1 ± 1.3 for those with higher BMIs (*p* = 0.32). 

In multivariable analysis adjusted for age, BMI, the number of LP attempts, and LP duration, significant pain and anxiety were not influenced by use of the active treatment (RR = 0.78 [0.41; 1.50], *p* = 0.46 and RR = 0.65 [0.34; 1.22], *p* = 0.18, respectively), whereas overall satisfaction (< or ≥9) was still associated with active treatment (RR = 2.15 [1.05; 4.43], *p* = 0.04).

Among the prespecified outcomes, the quality of blinding, evaluated using the Bang blinding index, was not perfect (index of 0.49 ± 0.09 vs. 0.33 ± 0.11, *p* < 0.001). 

Regarding the cost of this procedure, seven five-liter bottles (170 × 105 Pa 50%N_2_O-O_2_) were used to treat the 44 patients randomized to inhale 50%N_2_O-O_2_ during the whole study. Each bottle was purchased by the hospital pharmacy at a cost of €31.06. We also considered the cost of single-use masks (€0.80), as well as the one-way valve, filters, and pipes (€423.11 for 150 uses). In total, the additional cost of this procedure was a bit less than €8 per patient (including VAT). 

## 4. Discussion

In this trial, we show that a fixed 50% nitrous oxide/50% oxygen mixture effectively improved the overall satisfaction of the patients, with a very low NNT of only 2.4. Although the study did not reach its primary endpoint, as LP-induced pain was not significantly reduced using 50%N_2_O-O_2_ inhalation, the trend was clearly in favor of the active treatment, with an NNT of 5.5 for pain and 4.4 for anxiety. The adverse effects were mild and transient. The cost was limited to 8€ per patient. 

This study is the first double-blind randomized, controlled trial to compare a fixed 50%N_2_O-O_2_ mixture with placebo during an LP in an emergency setting. Contrary to a study performed in a neurology department [8], showing an NNT of 2.75 to avoid significant pain for one patient, the magnitude of the positive effect was not as large, leading to a statistically non-significant result. Several factors could explain this difference. First, in the study performed in the neurology department, LPs were planned. Contrary to those in the ED, the patients knew hours or days before the puncture that they would have an LP, mainly because of the difference of indications. Only 7.1% of our patients had already had an LP before our attempt, contrary to those in the neurology department (18.2%). Anxiety and pain before an LP are possibly more frequent in an emergency setting, and such parameters could have had a negative impact on the analgesic effect of the nitrous oxide [22]. Indeed, previous studies focusing on procedural pain [23,24,25,26,27] have consistently shown a positive effect, but these were not performed on adults in an emergency setting. Two studies performed on adults for moderate traumatic acute pain treatment in the ED were also positive [28,29]. Second, in the neurology department, LPs were performed by residents trained to perform such acts, reducing the overall number of LP attempts. In an ED, many different residents and physicians perform LPs, some having only limited experience for such a procedure. Such heterogeneity probably led to higher variability. Indeed, a study showed a higher failure rate of LPs for novices than experts [30] but no difference in pain intensity. Furthermore, LPs were performed during both day and night shifts by physicians and residents who had been potentially working for many hours, linked to an increased risk of serious medical errors [31,32]. Third, the patients were also more heterogeneous than in the previous study, with ages ranging from 18 to 89 years. In the present study, there was a correlation between the number of LP attempts and age. This association appears to be logical, in accordance with greater difficulty in performing LPs on older patients [33].

Although the magnitude of the efficacy could be questioned in light of our results, safety was good, with no major side effects reported. All side effects were those generally described for 50%N_2_O-O_2_ and were transient. Overall, the use of nitrous oxide is still debated due to a lack of efficacy in certain other medical situations [12] and frequent adverse effects in certain cases [13] and because some anesthesiologists believe that its use is obsolete [34]. This gas mixture has been used on a daily basis for over a century, but its mechanism of action is still not fully understood. NMDA antagonism has been demonstrated, but other mechanisms may also be involved [35]. Experimental studies have suggested that this immediate analgesic action is mediated by the release of endogenous opioids in the brainstem, particularly in the periaqueductal gray matter, leading to the activation of descending noradrenergic systems, but conflicting results have been obtained [36,37]. Despite its ease of use, 50%N_2_O-O_2_ is not often used during LP on adults. After a clearly positive trial for planned LPs [8] and a negative result in the present case, another large confirmatory study is ongoing (NCT03228628). This third study will be sufficiently large, with 162 planned patients, to confirm or reject the interest of the use of nitrous oxide for LP-induced pain and anxiety, at least in a neurology setting.

It has been shown that the BMI of the patient is the key factor that determines an unsuccessful LP [38]. In the study performed in neurology for scheduled LPs, secondary endpoints analyses identified a BMI > 25 kg/m^2^ as a risk factor for more severe pain during the procedure [8]. The present study did not confirm such an association. No other predictive factors regarding pain or anxiety were identified from the patient characteristics or responses to questionnaires before the LP. Thus, 50%N_2_O-O_2_ inhalation could be offered routinely to patients with no contraindication. Among these patients with varied profiles, the adverse effects observed were always mild and dissipated within minutes. 

Moving toward a pain-free hospital is, of course, a laudable objective. However, it remains very difficult in reality, especially in EDs [39,40,41,42]. As procedural pain is, by definition, predictable, this should be one of the priority targets to reduce patients’ pain. This study had several limitations. First, the trial was limited to a single center and there was a high diversity of residents and physicians performing the LPs. In addition, the expertise and seniority of those performing the LP were not analyzed. The high number of those performing LPs probably resulted in higher variability than in the first trial but is also more consistent with real-life practice in an ED. We did not study the duration of the wait between patient admission to the ED and the LP, nor the hour at which the LP was performed, which could possibly result in higher variability. Although negative for the primary outcome, our results still support the interest of this procedure as they reduced anxiety and increased overall patient satisfaction. Moreover, we did not include patients treated with only local anesthetics and not receiving any gas by inhalation as a control group. The placebo effect of medical air inhalation is probably quite high, reducing the effect size of the therapeutic gain of 50%N_2_O-O_2_ [43]. Of note, the usual practice in our institution is to use an EMLA patch to perform the cutaneous anesthesia, whereas the usual care in other hospitals consists of local anesthetics infiltration. Although both practices have proven effective [16], infiltration was not evaluated in the present study. It is also important to note that, sadly, many LPs are performed every day without any type of local anesthesia, particularly in EDs. The second limitation was the difficulty of maintaining perfect blinding with such a product that can modify sensory perception. The analysis we performed showed that although the bottles used were indistinguishable and perfumed masks were used, many patients correctly guessed the treatment (active or placebo) received. Third, although very safe for the vast majority of patients when used as 50%N_2_O-O_2_ for a short duration (usually less than 30 min for an LP), nitrous oxide can expose a patient to several risks. Indeed, methionine synthase antagonism induces vitamin B12 inactivation, which can lead to hematologic and neurologic complications. Such complications are not infrequent in the recreational use of nitrous oxide [44]. Nitrous oxide has also been shown to reduce parasympathetic tone [45]. It is also important to note that hypoxia, also known as diffusion hypoxia or the Fink effect, can occur during the washout of the gas, which should have been monitored and treated if present with 5 min inhalation of 100% oxygen. Thus, we suggest using pulse oximetry monitoring in daily practice. Fourth, the use of nitrous oxide has been reduced, also due to its environmental impact. Indeed, nitrous oxide is a greenhouse gas. Finally, the 50%N_2_O-O_2_ mixture is inexpensive compared to many other treatments used in high-income countries, but its cost may still limit its use in countries with a lower standard of living.

In conclusion, this trial demonstrates that 50%N_2_O-O_2_ inhalation has a limited effect for the management of immediate pain from lumbar puncture in an ED. However, it appears to be useful in reducing LP-induced anxiety and increases overall patient satisfaction. 

## Figures and Tables

**Table 1:** Demographic and baseline characteristics of the 84 patients with completed data, by randomization group. Four patients’ files were completely empty.

	Air (*n* = 41)	50%N_2_O-O_2_ (*n* = 43)	*p*-Value
Age (years), mean ± SD	47.2 ± 20.0	37.3 ± 15.3	0.02
Female sex, *n* (%)	23 (56.1)	27 (62.8)	0.53
BMI, mean ± SD	25.0 ± 4.7	24.4 ± 4.5	0.56
BMI, *n* (%)			
≥25	15 (34.1)	16 (36.4)	0.82
Previous LP, *n* (%)	2 (4.9)	4 (9.3)	0.68
LP indication, *n* (%)			0.09
Unusual headache	23 (56.1)	31 (72.1)	
Headache associated with fever	7 (17.1)	8 (18.6)	
Unexplained fever	5 (12.2)	0 (0.0)	
Others	6 (14.6)	4 (9.3)	
Analgesic drugs used, *n* (%)			0.80
NSAIDs	7 (17.1)	7 (16.3)	
Paracetamol	10 (24.4)	17 (39.5)	
Weak opioids	4 (9.8)	7 (16.3)	
Strong opioids	1 (2.4)	3 (7.0)	
Overall pain before LP	3.4 ± 2.8	3.9 ± 2.9	0.44
Anxiety before LP	5.0 ± 3.2	4.7 ± 3.4	0.70
Prior low back pain	10 (25.0)	12 (27.9)	0.76
LP duration (minutes), median [IQR]	15.0 [10.0–20.0]	10.5 [9.0–15.0]	0.12
Number of LP attempts, *n* (%)			0.07
1	12 (30.0)	25 (59.5)	
2	16 (40.0)	12 (28.6)	
3	6 (15.0)	2 (4.8)	
4	3 (7.5)	2 (4.8)	
5	3 (7.5)	1 (2.4)	

BMI: body mass index; IQR: interquartile range; LP: lumbar puncture; NSAIDs: Non-Steroidal Anti-Inflammatory drugs; SD: standard deviation.

**Table 2:** Summary of results concerning pain, anxiety, and patient satisfaction (intention-to-treat analysis).

	Air (*n* = 44)	50%N_2_O-O_2_ (*n* = 44)	SMD or AD	Statistics
Procedural pain (/10), mean ± SD Recorded 2-3 min after the end of gas inhalation	5.0 ± 2.9	4.2 ± 3.0	−0.27 [−0.69; 0.14]	*p* = 0.20
Procedural pain ≥ 4/10, *n* (%)	31 (70.5)	23 (52.3)	−0.18 [−0.38; 0.02]	*p* = 0.08
Procedural anxiety (/10), mean ± SD Recorded 2-3 min after the end of gas inhalation	4.7 ± 2.8	3.7 ± 3.7	−0.32 [−0.74; 0.09]	*p* = 0.13
Procedural anxiety ≥ 4/10, *n* (%)	32 (72.7)	22 (50.0)	**−0.23 [−0.43; −0.03]**	***p* = 0.03**
Overall satisfaction (/10), mean ± SD Recorded 1 h after the end of gas inhalation	7.4 ± 2.4	8.9 ± 1.6	**−0.73 [−0.30; −1.16]**	***p* < 0.001**
Overall satisfaction ≥ 9/10, *n* (%)	13 (29.6)	31 (70.5)	**−0.41 [−0.22; −0.60]**	***p* < 0.001**

SMD: standardized mean difference for continuous data; AD: absolute difference for categorical data; and 95% confidence interval. Negative values for SMD and AD indicated a difference in favor of the active group. Results with a *p*-value below 0.05 are noted in bold.

**Table 3:** Side effects described by the 78 patients with completed data, by randomization group. Only minor and transient side effects were reported.

	Air (*n* = 38)	50%N_2_O-O_2_ (*n* = 40)	*p*-Value
Nausea, *n* (%)	2 (5.3)	5 (12.5)	0.432
Paresthesia, *n* (%)	6 (15.8)	8 (20.0)	0.628
Sedation, *n* (%)	0 (0.0)	3 (7.5)	0.241
Changes in sensory perception, *n* (%)	2 (5.3)	14 (30.0)	0.002
Euphoria, *n* (%)	0 (0.0)	4 (10.0)	0.116
Restlessness, *n* (%)	1 (2.6)	2 (5.0)	1.000

**Table 4:** Predictive factors for LP-induced pain.

	Pain Score (NRS)	*p*-Value	NRS < 4	NRS ≥ 4	*p*-Value
	correlation coefficient				
Age (years)	−0.07	0.52	41.7 ± 16.0	41.9 ± 20.2	0.64
Number of LP attempts	0.39	**0.001**	1.4 ± 0.7	2.1 ± 1.2	**0.004**
LP duration	0.35	**0.002**	11.8 ± 6.1	15.7 ± 8.1	**0.02**
Overall pain before LP	0.15	0.19	3.6 ± 3.0	3.9 ± 2.7	0.66
Anxiety before LP	0.01	0.95	4.8 ± 3.6	5.1 ± 3.2	0.66
	mean ± sd				
Gender					0.24
Female (*n* = 47)	4.1 ± 3.2	0.21	23 (67.7)	24 (54.6)	
Male (*n* = 31)	4.9 ± 2.7		11 (32.3)	20 (45.4)	
BMI					0.29
<25 (*n* = 50)	4.3 ± 2.9	0.49	24 (70.6)	26 (59.1)	
25–35 (*n* = 28)	4.8 ± 3.2		10 (29.4)	18 (40.9)	
Prior low back pain					0.14
Yes (*n* = 21)	3.7 ± 3.0	0.11	12 (35.3)	9 (20.5)	
No (*n* = 57)	4.7 ± 3.0		22 (64.7)	35 (79.5)	

BMI: body mass index; NRS: numerical rating scale. Results with a *p*-value below 0.05 are noted in bold.

## Data Availability

The data presented in this study are available on request from the corresponding author.

## Supplementary Materials

The following supporting information can be downloaded at https://www.mdpi.com/article/10.3390/jcm11061489/s1, Table S1: Demographic and baseline characteristics of the 79 patients with completed data, by randomization group (per protocol analysis); Table S2: Summary of results concerning pain, anxiety, and patient satisfaction (per protocol analysis).
