# Supplementary material for: The Use of a Fixed 50:50 Mixture of Nitrous Oxide and Oxygen to Reduce Lumbar Puncture-Induced Pain in the Emergency Department: A Randomized Controlled Trial
Source: J Clin Med. 2022 Mar 9;11(6):1489. doi: 10.3390/jcm11061489 (PMC8953352; doi:10.3390/jcm11061489)
Supplement: Supplementary file 1 [file jcm-11-01489-s001.zip › jcm-1585407-supplementary.pdf]

Table S1. Demographic and baseline characteristics of the 79 patients with completed data, by randomization group (per protocol analysis).

	**Air** (***n*** **= 38)**	**50%N_2O-O_2** (***n*** **= 41)**	***p*-Value**
Age (years), mean ± SD	46.2 ± 20.2	37.3 ± 15.6	0.77
Female sex, *n* (%)	21 (55.3)	26 (63.4)	0.22
BMI, mean ± SD	24.7 ± 3.7	24.1 ± 4.1	0.26
BMI, *n* (%)			
≥25	14 (36.8)	14 (34.1)	0.80
Previous LP, *n* (%)	2 (5.3)	4 (9.8)	0.68
LP indication, *n (%)*			
Unusual headache	20 (52.6)	31 (75.6)	
Headache associated with fever	7 (18.4)	8 (19.5)	0.02
Unexplained fever	5 (13.2)	0 (0.0)	
Others	6 (15.8)	2 (4.9)	
Analgesic drugs used, *n* (%)			
NSAIDs	7 (18.4)	7 (17.1)	
Paracetamol	10 (26.3)	17 (41.5)	0.80
Weak opioids	4 (10.5)	7 (17.1)	
Strong opioids	1 (2.6)	3 (7.3)	
Overall pain before LP	3.6 ± 2.7	3.9 ± 3.0	0.71

Anxiety before LP	5.1 ± 3.2	4.8 ± 3.5	0.32
Prior low back pain	9 (23.7)	12 (29.3)	0.58
LP duration (minutes), median [IQR]	15.0 [10.0 – 20.0]	10.0 [8.5 – 15.0]	0.07
Number of LP attempts, *n* (%)			
1	12 (31.6)	25 (62.5)	
2	16 (42.1)	12 (30.0)	
3	4 (10.5)	1 (2.5)	
4	3 (7.9)	2 (5.0)	0.03
5	3 (7.9)	0 (0.0)	

BMI: body mass index; IQR: interquartile range; LP: lumbar puncture; NSAIDs: Non-Steroidal Anti-Inflammatory drugs; SD: standard deviation.

Table S2. Summary of results concerning pain, anxiety and patient satisfaction (per protocol analysis).

	Air (n = 38)	50%N_2O-O_2 (n = 40)	SMD or AD	Statistics
Procedural pain (/10), mean ± SD<br>Recorded 2–3 minutes after the end of gas inhalation	4.9±3.0	4.0±3.0	−0.29 [−0.73; 0.15]	*p*=0.20
Procedural pain ≥ 4/10, *n* (%)	25 (65.8)	19 (47.5)	−0.18 [−0.40; 0.03]	*p*=0.10
Procedural anxiety (/10), mean ± SD<br>Recorded 2–3 minutes after the end of gas inhalation	4.8±2.9	3.6±3.8	−0.36 [−0.80; 0.09]	*p*=0.12
Procedural anxiety ≥ 4/10, *n* (%)	27 (71.1)	19 (47.5)	**−0.24 [−0.45; −0.02]**	***p*=0.04**
Overall satisfaction (/10), mean ± SD<br>Recorded 1 hour after the end of gas inhalation	7.6±2.4	8.9±1.6	**−0.69 [−0.23; −1.14]**	***p*=0.003**
Overall satisfaction ≥ 9/10, *n* (%)	13 (34.2)	29 (70.7)	**−0.36 [−0.15; −0.57]**	***p*=0.001**

SMD: standardized mean difference for continuous data, AD: absolute difference for categorical data, and 95% confidence interval. Negative values for SMD and AD indicated a difference in favor of active group. Results with a p-value below 0.05 are noted in bold.
